# Differential expression and effect analysis of lncRNA-mRNA in congenital pseudarthrosis of the tibia

**DOI:** 10.3389/fgene.2023.1094298

**Published:** 2023-02-06

**Authors:** Zhuoyang Li, Haibo Mei, Kun Liu, Ge Yang

**Affiliations:** ^1^ Department of Orthopedics, The First Affiliated Hospital, College of Medicine, Zhejiang University, Hangzhou, China; ^2^ Department of Orthopedics, Hunan Children’s Hospital, Changsha, Hunan, China

**Keywords:** congenital pseudarthrosis of the tibia, long non-coding RNA, messenger RNA, periosteal stem cell, bioinformatic analysis

## Abstract

**Background:** To analyze the lncRNA-mRNA differential expression and co-expression network of periosteal stem cells (PSCs) from congenital pseudarthrosis of the tibia (CPT) and normal patients, and to explore the role of key lncRNAs.

**Methods:** Differentially expressed lncRNAs and mRNAs in PSCs were obtained by sequencing, and biological functions of differentially expressed mRNAs were detected by gene ontology (GO), Kyoto encyclopedia of genes and genomes (KEGG) pathway and protein -protein interaction (PPI) analysis. The co-expression network of lncRNA-mRNA was constructed by correlation analysis of differentially expressed lncRNAs and mRNAs, and the key lncRNAs were screened according to the connectivity degree. After that, the cis-regulated target genes of differential expressed lncRNAs and mRNAs were predicted.

**Results:** A total of 194 differentially expressed lncRNAs were identified, including 73 upregulated and 121 downregulated genes. A total of 822 differentially expressed mRNAs were identified, including 311 upregulated and 511 downregulated genes. GO, KEGG and PPI enrichment analysis showed that the regulatory function of differentially expressed mRNAs were mainly gathered in skeletal system development and tissue morphogenesis. The co-expression network with 226 nodes and 3,390 edges was constructed based on correlation analysis. A total of 10 key lncRNAs, including FAM227B, POM121L9P, AF165147 and AC103702, were screened according to connectivity degree. Prediction of target genes indicated that FAM227B-FGF7 and AC103702-HOXB4/5/6 may play an important role in the pathogenesis of CPT.

**Conclusion:** A total of 10 key lncRNAs, including FAM227B, POM121L9P, AF165147, and AC103702, occupy the core position in the co-expression network, suggesting that these lncRNAs and their target genes may play an important role in the pathogenesis of CPT.

## 1 Introduction

Congenital pseudarthrosis of the tibia (CPT) is regarded as a rare, refractory and long-term congenital disease in children. It is reported that the incidence rate of CPT ranges from about 1: 140,000 to 1: 250,000 (Hefti et al., 2000). Since Paget reported the disease in 1891, its pathogenesis remains to be unclear (BOYDHarold, 1982; Pannier, 2011). Currently, surgical treatment is the main treatment for CPT patients in clinic. However, according to the literatures, the risk of complications such as bone non-union, re-fracture, limb length difference and ankle valgus after CPT can reach 50% (Li et al., 2022), which results in reoperation, as well as even severe consequences such as disability and amputation (Zhou et al., 2022) and brings huge psychological and economic burden to patients and their families. Therefore, it is the key to seek more effective treatment through deeply studying the pathogenesis of CPT.

Previous studies have found that tibial periosteum lesion is an important pathogenic factor causing the decline of local bone osteogenesis, which results in the formation of CPT. Therefore, it has gradually become a hot spot to explore the biological characteristics of periosteum lesion in CPT. Periosteum is a connective tissue membrane which covers the bone surface and possesses rich micro-vascular tissue. It has good osteogenic ability and plays an important role in bone growth, development and injury repair (Filion et al., 2017). Periosteum is mainly composed of three layers: fibrous layer, undifferentiated layer and germinal layer. Germinal layer is composed of periosteal stem cells (PSCs), mesenchymal stem cells, fibroblasts and other cells (Augustin et al., 2007). Recent studies have confirmed that periosteal stem cells in germinal layer have excellent osteogenic potential, and they can differentiate into osteoblasts under specific conditions, which are important functional cells involved in the process of bone remodeling and repair (Doherty et al., 2020; Matthews Brya et al., 2021). The osteogenic differentiation ability of PSCs is affected by many factors, such as local micro-environment (various hormones, cytokines, transcription factors) (Granchi et al., 2010), but the specific ways to affect the osteogenic differentiation ability of PSCs still remain to be unknown.

In recent years, long-chain non-coding RNA (lncRNA) has been the hot spot of gene expression regulation. LncRNA is a non-coding RNA composed of more than 200 nucleotides (Miao and Lee, 2015). Initially lncRNA was considered as noise in the transcription process, without biological function (Iyer Matthew et al., 2015). However, more and more studies have shown that lncRNA is closely related to many physiological activities and pathological processes. Some studies reported that lncRNA participates in the process of regulating many common diseases, such as various cancers, cardiovascular diseases, metabolic diseases (Pearson et al., 2016; Mila and Alessia, 2019; Macdonald and Mann, 2020). With the development of technology, more and more scholars found that lncRNA is also related to some rare diseases, such as Prader-Willi syndrome (Wu et al., 2016), but there are still few studies on CPT.

In order to better understand the pathogenesis of CPT and explore the clues of the key pathogenic genes, we used the lncRNA microarray technology to detect the differences in lncRNA and messenger RNA (mRNA) expression profiles of PSCs between normal people and CPT patients. In addition, the function and interaction of lncRNAs and mRNAs were comprehensively analyzed, aiming to provide experimental basis for finding new therapeutic targets for CPT.

## 2 Materials and methods

### 2.1 Sample acquisition

The samples of this study came from patients who were admitted into the department of Orthopaedics of The First Affiliated Hospital of Zhejiang University, School of Medicine and Hunan Children’s Hospital from 2018 to 2020. Normal periosteal tissues were obtained from the operation of healthy children with tibial fracture caused by trauma in The First Affiliated Hospital of Zhejiang University, School of Medicine, and the diseased periosteum tissues were obtained from CPT patients during surgery in Hunan Children’s Hospital. A total of 10 periosteum samples were obtained and divided into two groups: control group and CPT group, with five from CPT patients (3–8 years old) and five from traumatic children (3–6 years old) separately. This study had been approved by the Ethics Committee of the First Affiliated Hospital of Zhejiang University, School of Medicine and Hunan Children’s Hospital, and informed consent had been obtained from all patients.

### 2.2 Isolation, purification and identification of PSCs

After the periosteal tissue sample was cut to 1 mm^3^, it was digested at 37°C for 12 min with collagenase A (2 mg/mL, Roche) and trypsin (2.5 mg/mL, Roche). After DMEM culture solution containing 10% FBS was added, the supernatant was removed by centrifugation. DMEM culture solution containing 10% FBS was added, and the tissue was resuspended gently for 5–10 times. The suspension was filtered by a 70-um cell sieve, and the filtrate was inoculated into a culture dish. When the cell confluence reached 90%–100%, the cells were digested by trypsin for subculture. STRO-1 was used as marker, and PSCs were purified by indirect immunomagnetic bead positive selection. DSB-X Botin STRO-1 labeled antibodies were co-incubated with PSCs at 4°C for 30 min, and the supernatant was removed after centrifugation at 1000 rpm for 8 min 80 ul of magnetic beads and PSCs were mixed for 30 min and put them in the magnetic field for 3 min to remove the supernatant. Flowcomp release buffer was added. After repeated mixing, magnetic field was placed to demagnetize the beads so as to obtain purified PSCs. Fluorescently labeled monoclonal antibodies CD31, CD34, CD44, and CD90 were added to the purified PSCs, and the mixture was mixed and left for 15 min at 4°C. After washing 3 times with PBS, 500 ul of PBS containing 0.5% FBS was added and transferred to a flow cytometer for detection.

### 2.3 RNA extraction library construction and sequencing

**FIGURE 1:**
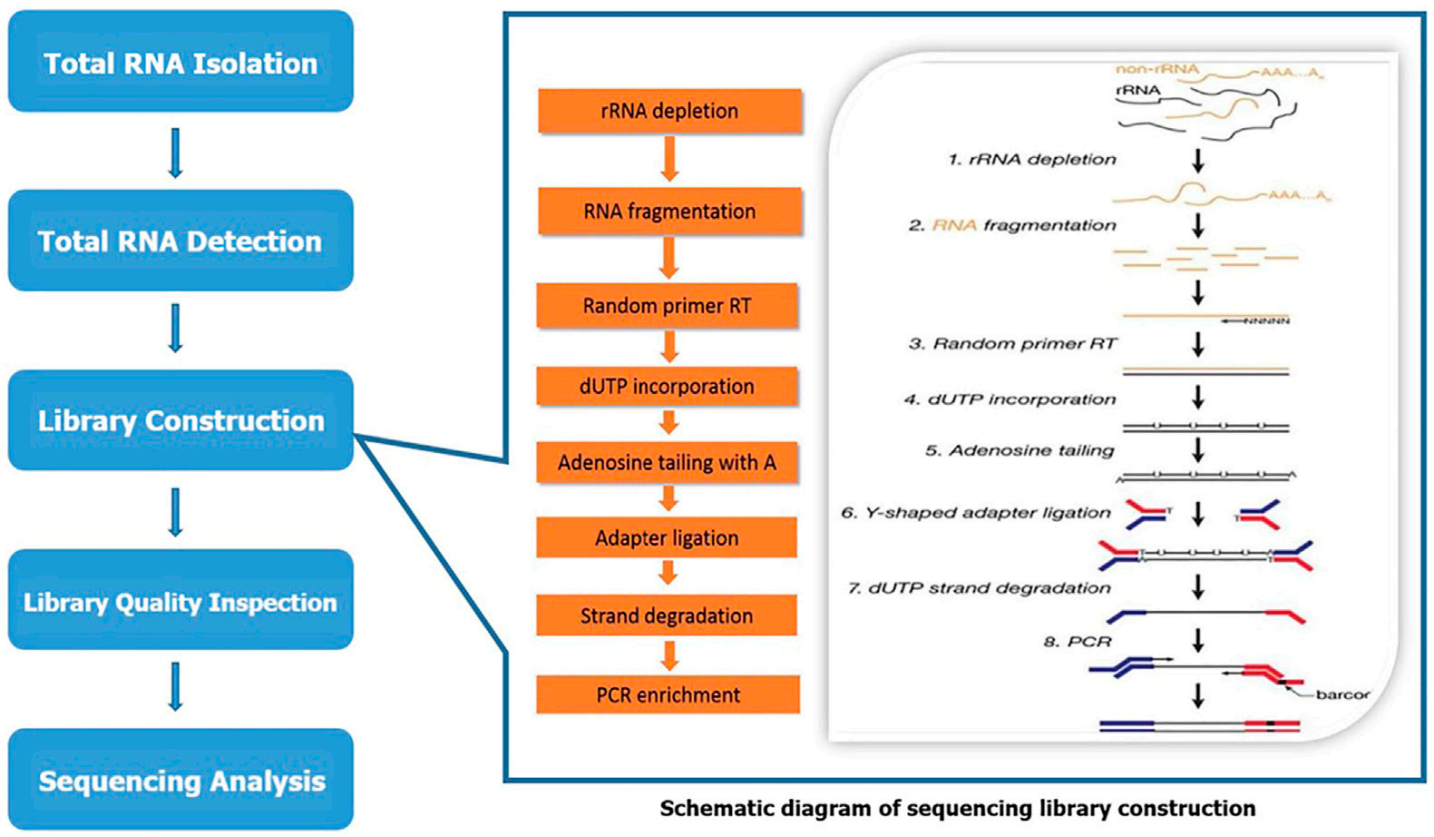
The schematic diagram of sequencing.

LC bio-technology Co., Ltd. (Hangzhou, China) provided microarray technical support for analyzing and evaluating lncRNA and mRNA expression profiles. The microarray sequencing process is shown in Figure 1. Total RNA was extracted using Trizol reagent (hermofisher, 15596018) following the manufacturer’s procedure. The total RNA quantity and purity were analyzed of Bioanalyzer 2100 and RNA 6000 Nano LabChip Kit (Agilent, CA, United States, 5067-1511) with RIN number >7.0. Approximately 5 ug of total RNA was used to deplete ribosomal RNA according to the manuscript of the Ribo-Zero Gold rRNA Removal Kit (Illumina, cat. MRZG12324, San Diego, United States). After removing ribosomal RNAs, the left RNAs were fragmented (NEBNext^®^ Magnesium RNA Fragmentation Module, cat. E6150S, United States) into short fragments using divalent cations under high temperature. Then the cleaved RNA fragments were reverse transcribed to create the cDNA by SuperScript™ II Reverse Transcriptase (Invitrogen, cat. 1896649, United States), which were next used to synthesise U-labeled second-stranded DNAs with *E. coli* DNA polymerase I (NEB, cat. m0209, United States), RNase H (NEB, cat. m0297, United States) and dUTP Solution (Thermo Fisher, cat. R0133, United States). An A-base was then added to the blunt ends of each strand, preparing them for ligation to the indexed adapters. Each adapter contains a T-base overhang for ligating the adapter to the A-tailed fragmented DNA. Single- or dual-index adapters are ligated to the fragments, and size selection (300–600 bp) was performed with AMPureXP beads. After the heat-labile UDG enzyme (NEB, cat. m0280, United States) treatment of the U-labeled second-stranded DNAs, the ligated products were amplified with PCR by the following conditions: initial denaturation at 95°C for 3 min; 8 cycles of denaturation at 98°C for 15 s, annealing at 60°C for 15 s, and extension at 72°C for 30 s; and then final extension at 72°C for 5 min. The average insert size for the final cDNA library was 300 ± 50bp. At last, we performed the 2 × 150 bp paired-end sequencing (PE150) on an Illumina Novaseq™ 6000 (LC Bio-Technology CO. Ltd. Hangzhou, China) following the vendor’s recommended protocol.

### 2.4 Bioinformatics analysis

#### 2.4.1 Different expression analysis of mRNAs and lncRNAs

Genes/RNAs differential expression analysis was performed by DESeq2software (Smyth and McCarthy, 2010; Pertea et al., 2015) between two different groups (and by edgeR between two samples). The genes/mRNA/lncRNA with the parameter of false discovery rate (FDR) below 0.05 and absolute fold change ≥2 were considered differentially expressed genes/mRNA/lncRNA. Differentially expressed coding RNAs were then subjected to enrichment analysis of gene ontology (GO) functions and Kyoto encyclopedia of genes and genomes (KEGG) pathways. Cluster analysis was carried out and plotted by heat map, volcanic map.

#### 2.4.2 GO enrichment analysis

GO (http://www.geneontology.org/) is an international standardized gene functional classification system which offers a dynamic-updated controlled vocabulary and a strictly defined concept to comprehensively describe properties of genes and their products in any organism. GO has three ontologies: molecular function, cellular component and biological process. The basic unit of GO is GO-term. Each GO-term belongs to a type of ontology. GO enrichment analysis provides all GO terms that significantly enriched in DEGs comparing to the genome background. Firstly, all DEGs were mapped to GO terms in the Gene Ontology database, gene numbers were calculated for every term, significantly enriched GO terms in DEGs comparing to the genome background were defined by hypergeometric test and then ranked according to the -log10 (p). The calculating formula of *p*-value is:

$$P=1-\overset{m-1}{\underset{i=0}{\sum }}\frac{\left(\begin{array}{l} M\\ i \end{array}\right)\left(\begin{array}{l} N-M\\ n-i \end{array}\right)}{\left(\begin{array}{l} N\\ n \end{array}\right)}$$

Here N is the number of all genes with GO annotation; n is the number of DEGs in N; M is the number of all genes that are annotated to the certain GO terms; m is the number of DEGs in M. GO terms meeting this condition with *p* < 0.05 were defined as significantly enriched GO terms in DEGs. Significant enriched GO terms were then hierarchically clustered into a tree based on Kappa-statistical similarities among their gene memberships. 0.3 Kappa score was applied as the threshold to cast the tree into term clusters. This analysis was able to recognize the main biological functions that DEGs exercise.

#### 2.4.3 Pathway enrichment analysis

Genes usually interact with each other to play roles in certain biological functions. Pathway-based analysis helps to further understand genes biological functions. KEGG (www.genome.jp/kegg) is a public database on genome deciphering. The databases such as GENES and Pathway contain information of cellular biochemical processes, including genome sequence, metabolism, membrane transport, chemical substances and enzyme reactions. In organisms, different genes coordinate their biological functions, and pathway enrichment analysis identified significantly enriched metabolic pathways or signal transduction pathways in DEGs comparing with the whole genome background. The calculating formula is the same as that in GO analysis. Here N is the number of all genes that with KEGG annotation, n is the number of DEGs in N, M is the number of all genes annotated to specific pathways, and m is number of DEGs in M. Pathways meeting this condition with *p* < 0.05 were defined as significantly enriched pathways in DEGs.

#### 2.4.4 Protein -protein interaction (PPI) network analysis

String software (http://string-db.org) was used to analyze the interaction between target genes encoded by differentially expressed lncRNA-mRNA and corresponding proteins, and the key proteins were screened out. Minimum required interaction score >0.7 was set to obtain the interactive relationship between proteins and construct the PPI network. MCODE (Molecular Complex Detection) algorithm was then applied to this network to identify neighborhoods where proteins are densely connected. GO enrichment analysis was applied to each MCODE network to extract “biological meanings” from the network component, where top three best *p*-value terms were retained. Cytoscape software was imported for visualization.

#### 2.4.5 LncRNA-mRNA co-expression network

The differentially expressed lncRNA-mRNA co-expression network was constructed to explore their interaction in the of normal and CPT patients. By calculating Pearson’s correlation coefficient (r) of lncRNAs and mRNAs, the correlation of their expression levels was obtained. The significantly correlated lncRNA-mRNA pairs were screened by setting |r| > 0.9 and *p* < 0.01 for constructing lncRNA-mRNA co-expression network, and then Cytoscape software was imported for visualization. Connectivity degree is an index used to evaluate the importance of lncRNA-mRNA in the network. The greater degree is, the greater its regulation effect on the network is. By calculation, the top 10 differentially expressed lncRNAs were listed.

#### 2.4.6 Prediction of lncRNA target gene

The main regulation modes of lncRNA include cis-regulation and trans-regulation (Tachiwana et al., 2020). The lncRNA cis-regulation target genes were mainly predicted according to the positional relationship. The differentially expressed lncRNA and mRNA within 100 kbp upstream and downstream of chromosome were defined as cis regulation. Differential lncRNAs and mRNAs in cis-regulation were predicted and analyzed.

### 2.5 Quantitative reverse transcription-polymerase chain reaction (qRT-PCR)

We selected the top five differentially expressed lncRNAs mentioned above which were verified by qRT-qPCR. The analysis was performed according to the instructions: the cells were lysed, and RNA was extracted with Trizol reagent (Invitrogen, 15596018). The EntiLink™first strand cDNA Synthesis Kit (ELK Biotechnology, EQ003) was used to synthesize the first strand cDNA. qRT-PCR was performed using an ABI Quant Studio six Real-Time PCR instrument (Life technologies). Three double Wells were made for each sample and SYBR Green PCR Super Mix kit (VAZYME, Q111-02) was used. The reaction conditions were 95°C (15 s), 60°C (60 s), 95°C (15 s), and 40 cycles. The quantitative method was 2^^−ΔΔ^ CT method. The primer sequences are shown in Table1.

**TABLE 1:** The primer sequences.

Primer	Primer Sequence (5′-3′)
*FAM227B*	GCT​CTT​TTG​CAT​GAC​TCC​TTT​TG
	TCC​TTT​CGA​CTT​AGA​GGT​ATG​CT
*POM121L9P*	CCG​GAA​CGA​AAG​TGG​AAG​AGG
	ATC​TGC​CGT​CTT​TTC​ACA​CTG
*TRAID*	ATC​TCA​TCA​AGC​CCT​CTG​TAG​T
	GGA​CGC​AGG​TCA​TGG​AAG​C
*SRP9*	CTT​TAC​CTC​GCT​GAC​CCT​ATG​A
	GTC​GCA​TTA​GTT​GAC​TGT​GGA​A
*AF165147*	GCA​GGG​GAG​AAC​TTC​GCT​AC
	CAG​CGC​GTT​CAA​AAG​CAG​AAA

## 3 Results

### 3.1 Differential lncRNA and mRNA screening

**FIGURE 2:**
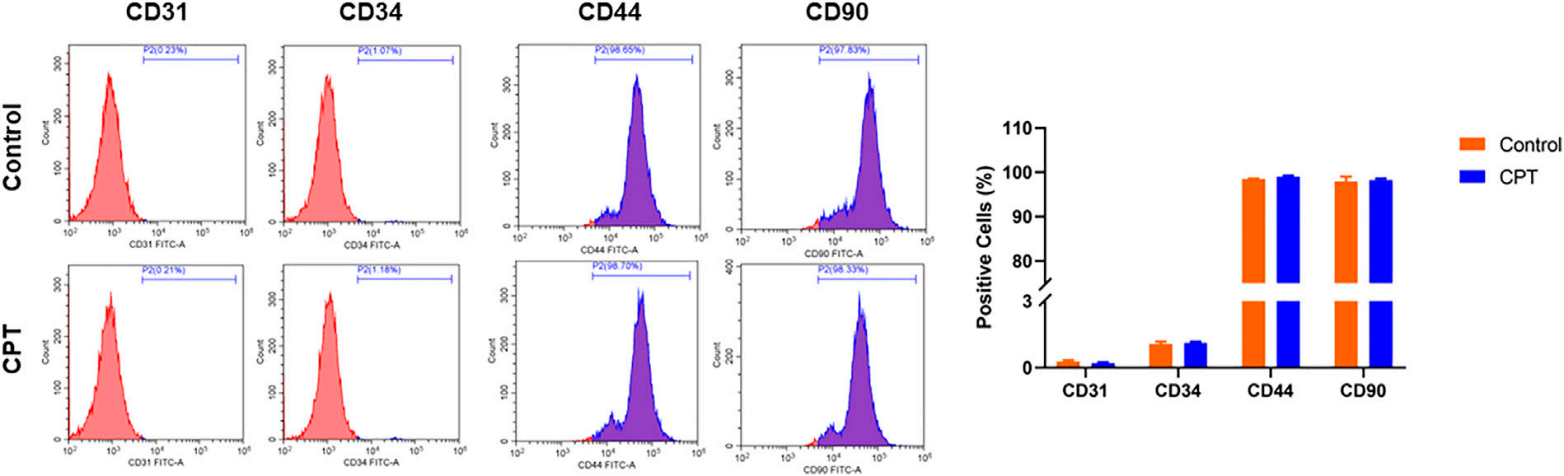
Flow cytometry was used to detect the positive ratio of molecular markers CD31, CD34, CD44 and CD90 of purified PSCs derived from different periosteal tissues. PSCs, periosteal stem cells.

The normal and CPT periosteal cells were isolated and purified through the above steps, and the purified cell surface markers were identified by flow cytometry. The results showed that the average positive rate of the identified cell lines CD31 and CD34 was lower than 1%, and the average positive rate of CD44 and CD90 was higher than 98%, which was identified as PSCs (Figure 2).

Further, we used the purified PSCs for sequencing analysis. According to the screening criteria (absolute fold change ≥2), 194 differentially expressed lncRNAs were identified by sequencing, including 73 upregulated genes and 121 downregulated genes, with 116 newly discovered lncRNAs and 78 known lncRNAs. There were 822 differentially expressed mRNAs, including 311 upregulated genes and 511 downregulated genes.

**FIGURE 3:**
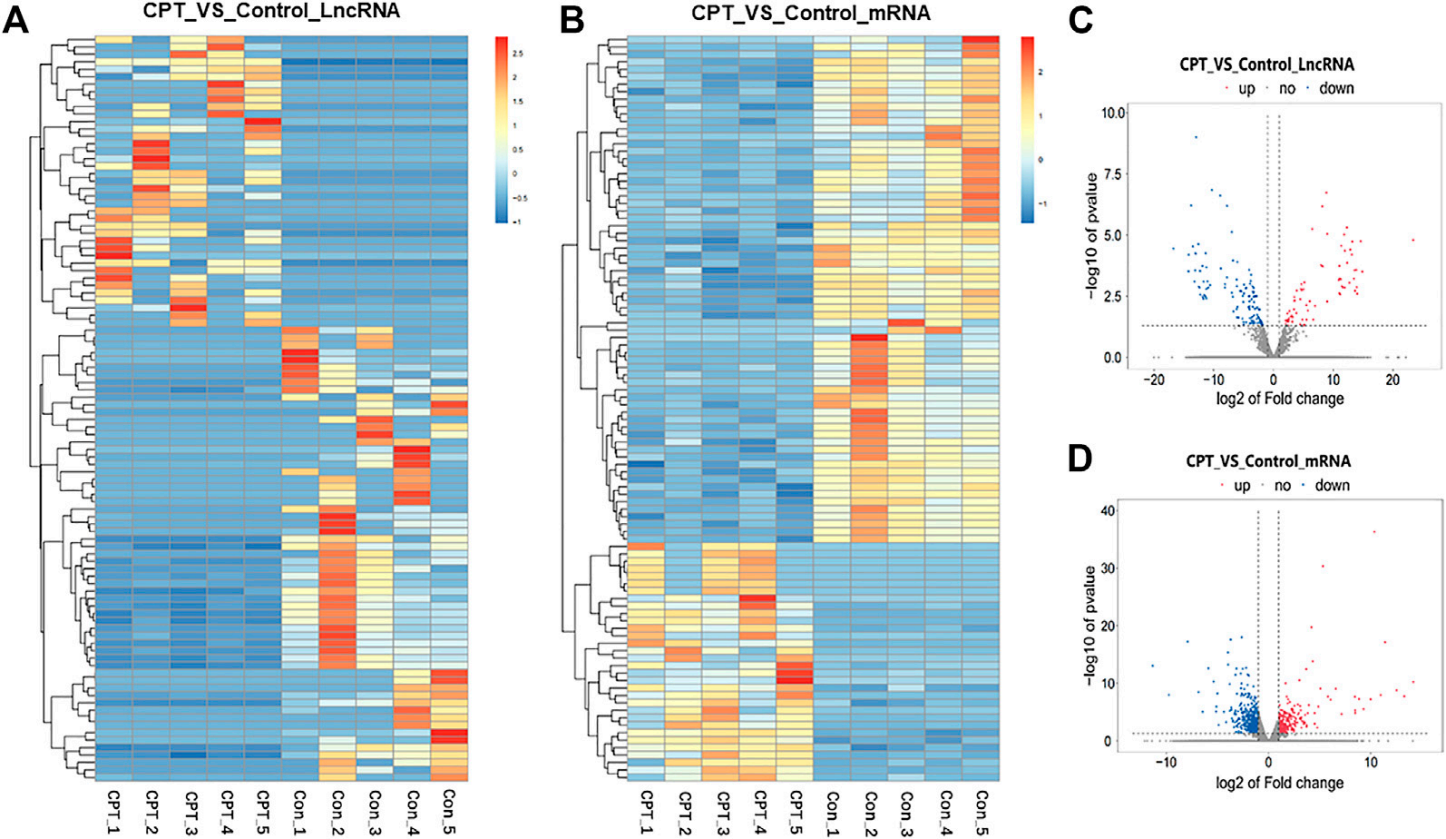
**(A)**, the first 100 lncRNAs with the smallest *p*-value were plotted by heat map. **(B)**, the first 100 mRNAs with the smallest *p*-value were plotted by heat map. **(C)**, the expression difference of lncRNA in normal and CPT PSCs was analyzed by volcanic map. **(D)**, the expression difference of mRNA in normal and CPT PSCs was analyzed by volcanic map. CPT, congenital pseudarthrosis of the tibia; PSCs, periosteal stem cells.

At the same time, cluster analysis was carried out, and the first 100 lncRNAs and mRNAs with the smallest *p*-value were plotted by heat map (Figures 3A, B). Moreover, the expression difference of lncRNA and mRNA in normal and CPT PSCs were analyzed by volcanic map (Figures 3C, D).

### 3.2 Gene function annotation and pathway enrichment analysis

**FIGURE 4:**
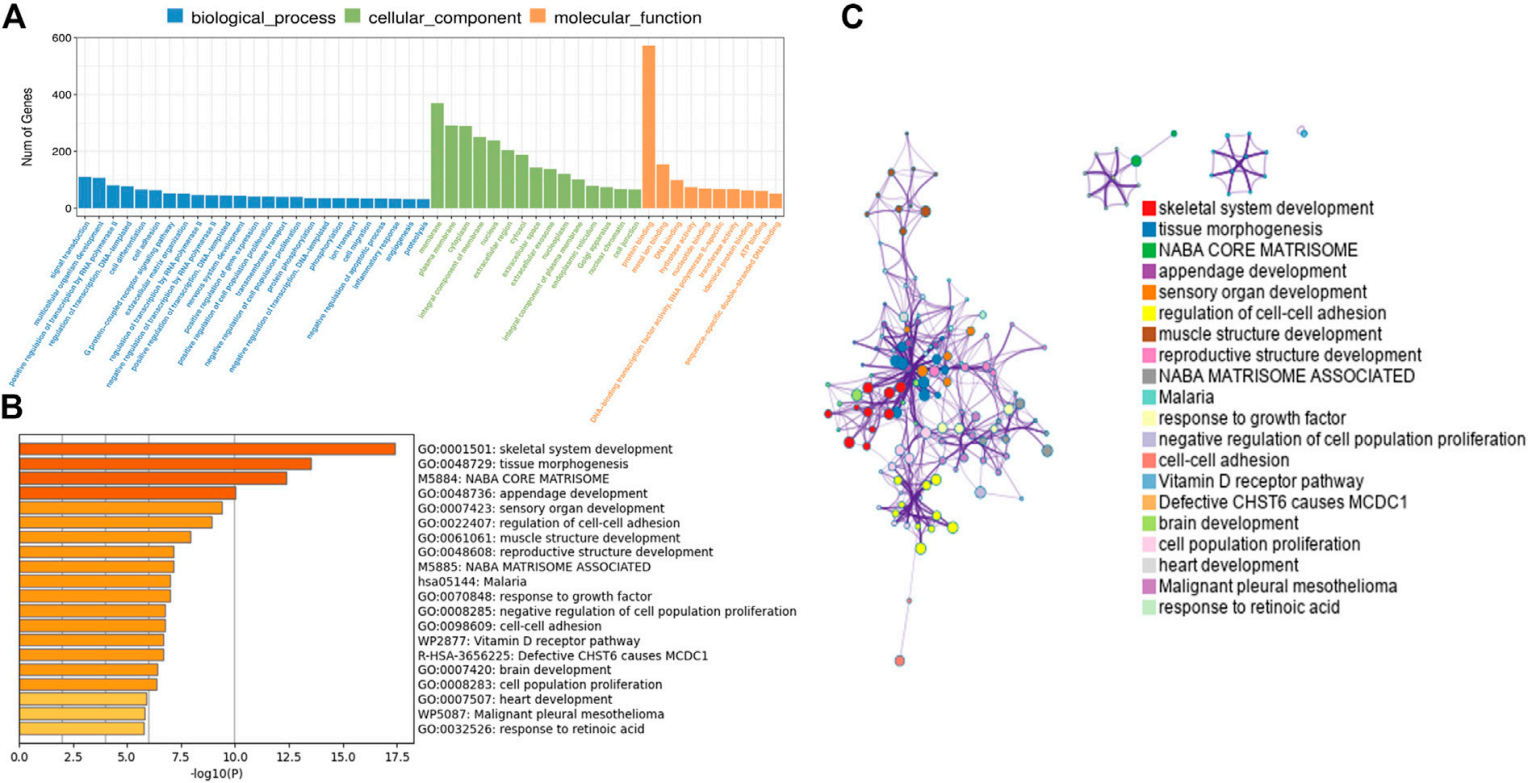
**(A)**, GO analysis revealed gene enrichment in different cellular biological processes. **(B)**, the top 20 significant GO terms were hierarchically clustered into a tree based on Kappa-statistical similarities among their gene memberships. **(C)**, the representative terms from the full cluster were selected and converted into a network layout. Each term was represented by a circle node, where its size is proportional to the number of genes fall under that term, and its color represent its cluster identity (nodes of the same color belong to the same cluster). Terms with a similarity score >0.3 were linked by an edge (the thickness of the edge represents the similarity score). GO, Gene ontology.

In order to further analyze the function of differentially expressed mRNAs in normal and CPT PSCs, GO and KEGG pathway analysis were carried out. GO analysis showed that the biological processes of differentially expressed mRNAs was mainly focused on signal transduction, multicellular organism development and positive regulation by transcription RNA polymerase II; secondly the cell components were concentrated in cell membrane, plasma membrane and cytoplasm; thirdly, molecular biological functions were focused on protein binding, metal ion binding and DNA binding, etc (Figure 4A). Significant enriched GO terms were then clustered based on Kappa-statistical similarities among their gene memberships. The results showed that the biological functions with the most significant differences were mainly gathered in skeletal system development, tissue morphogenesis and NABA core matrisome (shown in Figures 4B, C).

**FIGURE 5:**
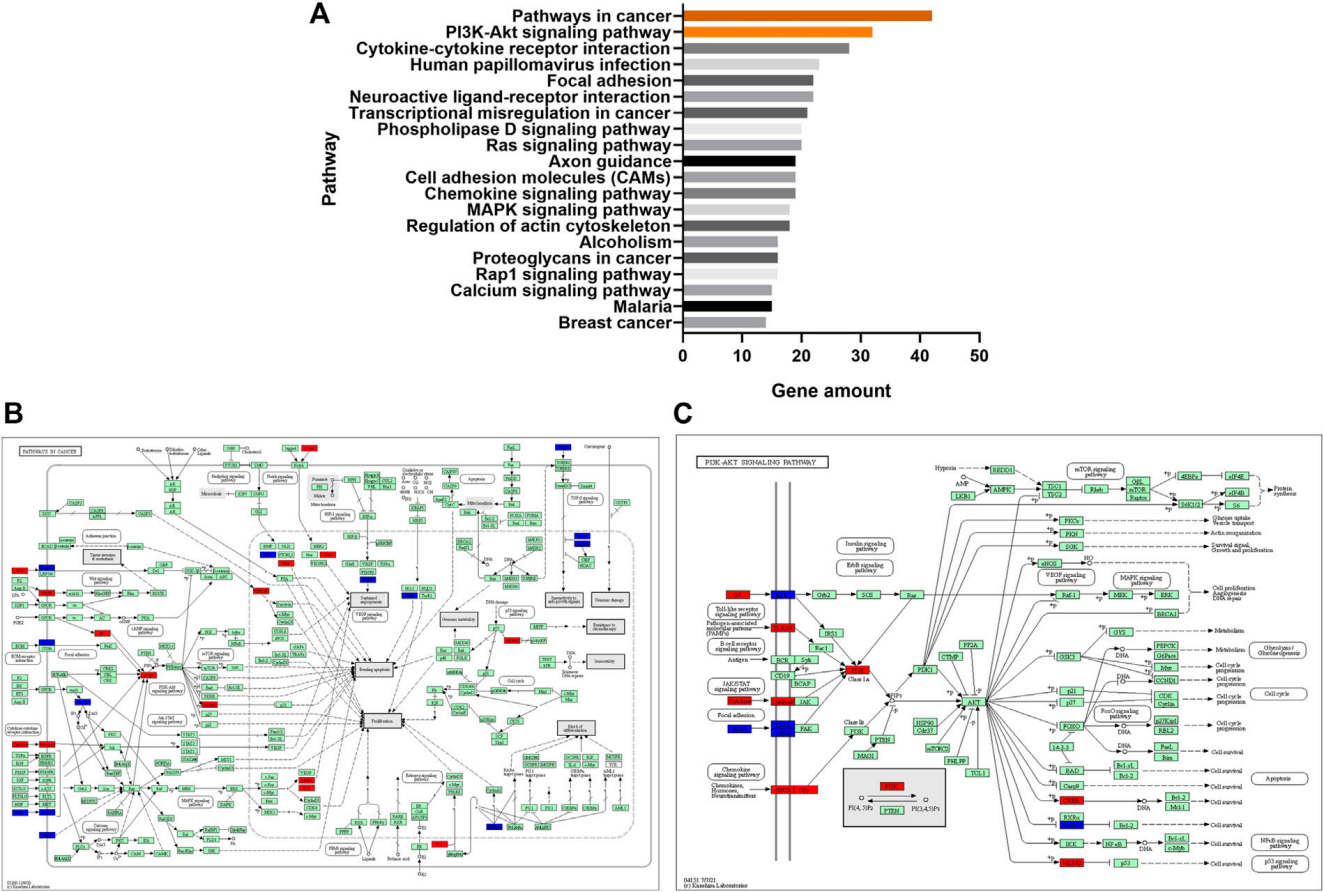
**(A)**, the top 20 significant signaling pathways were hierarchically clustered into a tree, which showed that the pathways with the most significant differences were mainly concentrated in pathways in cancer and PI3K-Akt signaling pathway. **(B)**, the regulatory changes of related mRNAs involved in the pathways of cancer. Red represented upregulation and blue represented downregulation. **(C)**, the regulatory changes of related mRNAs involved in PI3K-Akt signaling pathway. Red represented upregulation and blue represented downregulation.

The KEGG pathways enrichment analysis showed that 59 pathways had significant difference (*p* < 0.05), with 30 upregulated pathways and 29 downregulated pathways. A total of 20 pathways with the most significant regulation were selected. KEGG analysis showed that the pathways with the most significant differences were mainly concentrated in pathways in cancer, PI3K-Akt signaling pathway and cytokine-cytokine receptor interaction (Figure 5A). The regulatory changes of related mRNAs involved in the pathways of cancer and PI3K-Akt signaling pathway were shown in Figures 5B, C.

### 3.3 PPI network analysis

**FIGURE 6:**
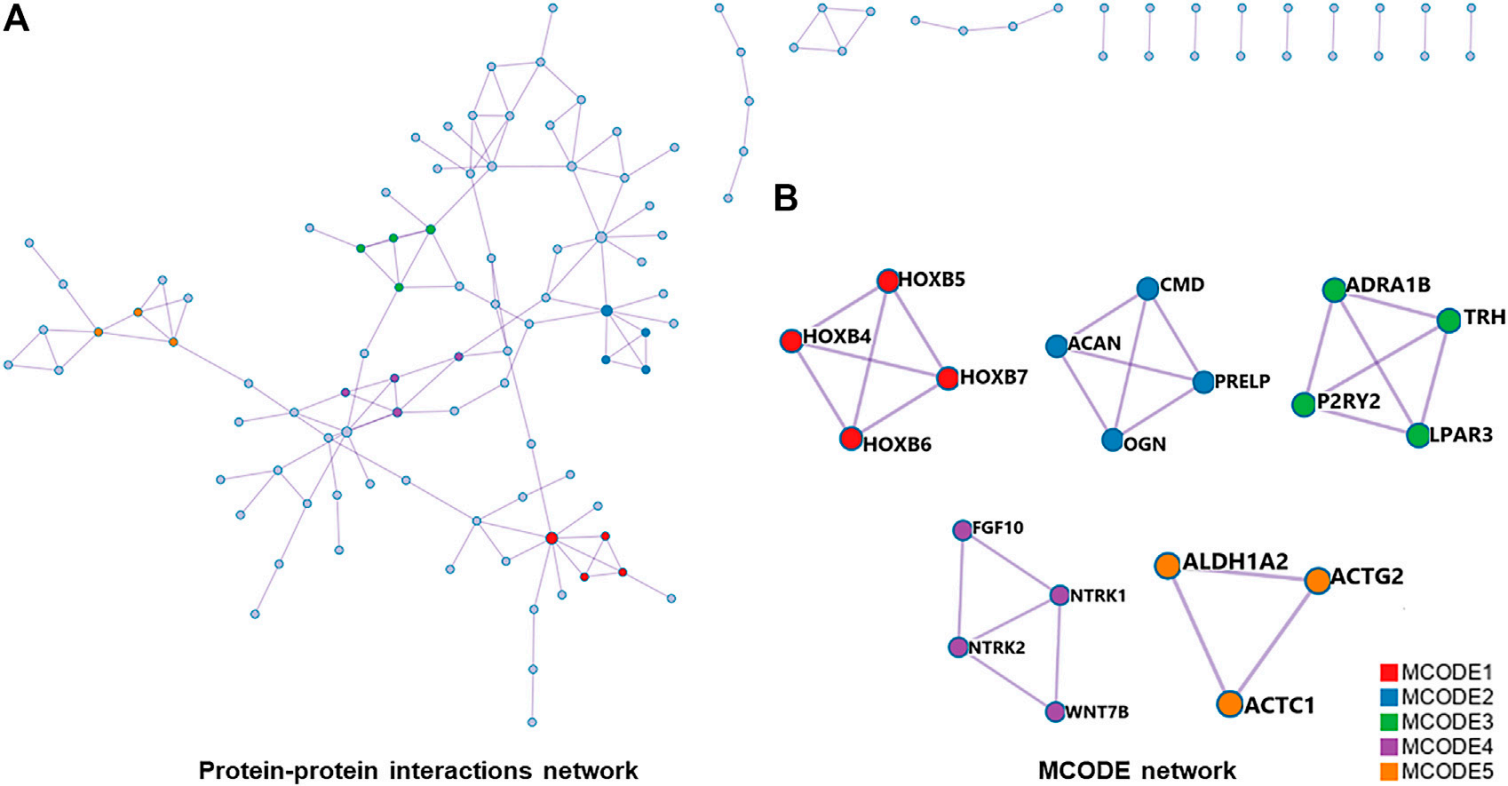
**(A)**, all protein-protein interactions among differentially expressed mRNAs were extracted from PPI data source and formed a PPI network. **(B)**, MCODE algorithm was applied to identify neighborhoods where proteins were densely connected. Five MCODE modules were enriched. PPI, protein-protein interaction; MCODE, Molecular Complex Detection.

For differentially expressed mRNAs, protein-protein interaction enrichment analysis was carried out through String (Damian et al., 2019). MCODE (Bader and Hogue, 2003) was also used to identify closely connected network components, and five functional components with the highest *p*-value were obtained. The PPI included 118 nodes and 144 interactions (Figure 6A). According to the annotation of MCODE, PPI enrichment analysis showed that five MCODE modules were enriched (Figure 6B) and the regulatory function of differentially expressed mRNAs were mainly concentrated in skeletal system development, tissue morphogenesis, MAPK signaling pathway regulation, etc (shown in Supplementary Table S1).

### 3.4 Co-expression network and target prediction of lncRNA-mRNA

**FIGURE 7:**
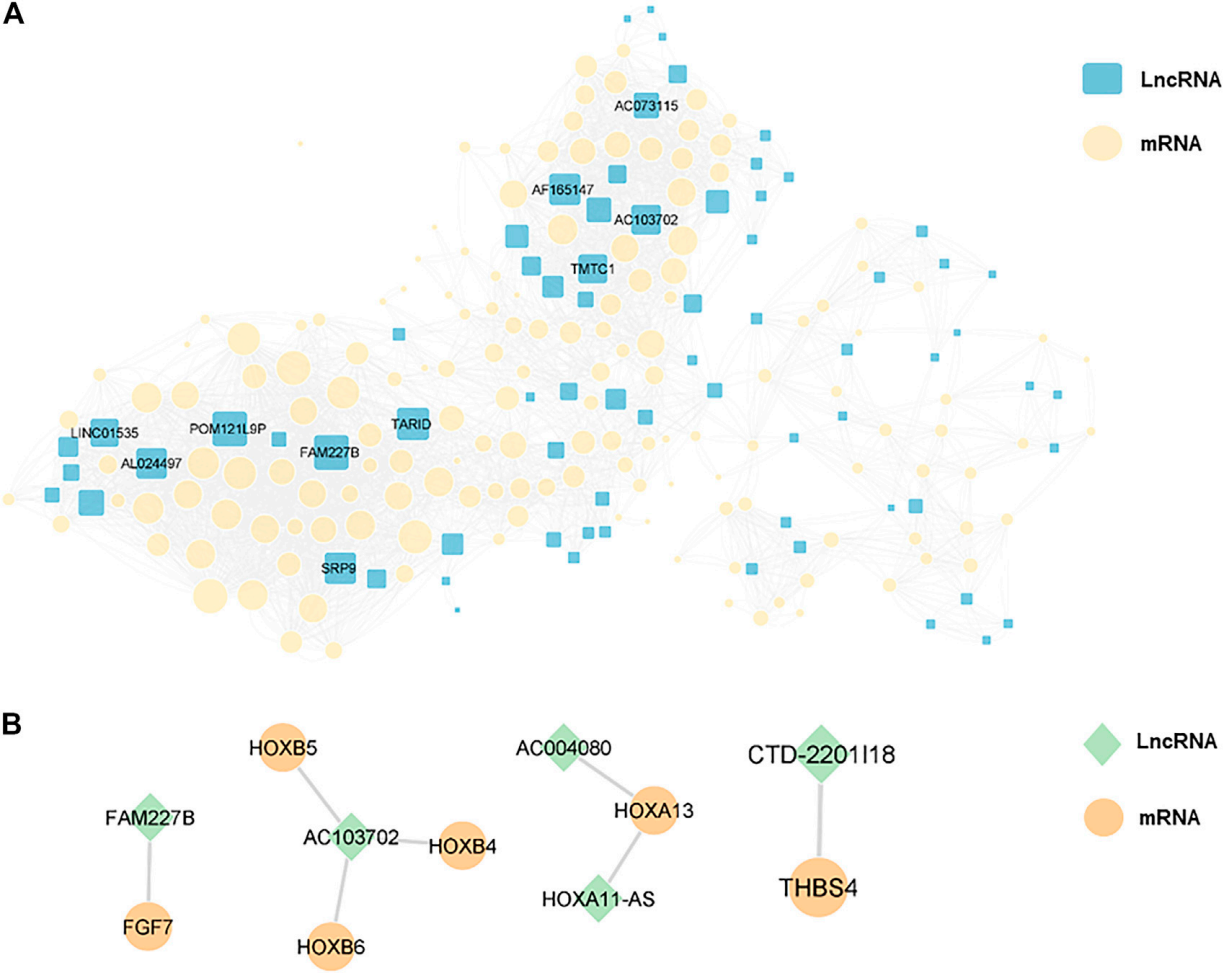
**(A)**, a lncRNA-mRNA co-expression network with 226 nodes and 3,390 edges was constructed, and the top 10 lncRNAs ranked by connectivity degree in the network were highlighted as squares. The larger the square, the higher connectivity degree. Blue squares represented lncRNAs and yellow circles represented mRNAs. **(B)**, cis-regulation predictive analysis on the differentially expressed lncRNAs and mRNAs was performed. The top five lncRNAs with significant differences were listed separately, with green diamonds represented lncRNAs and yellow circles represented mRNAs.

In order to explore the interaction between differentially expressed lncRNAs and mRNAs, we constructed a lncRNA-mRNA co-expression network with 226 nodes and 3,390 edges, according to |r| > 0.9 and *p* < 0.01 (Figure 7A). Moreover, we sorted the differentially expressed lncRNAs by connectivity degree, and the top 10 lncRNAs were listed in Table 2, such as FAM227B, POM121L9P, AF165147, AC103702, for further study.

**TABLE 2:** The top 10 lncRNAs were sorted by connectivity degree.

Number	Gene	Degree	Regulation
1	FAM227B	74	Down
2	POM121L9P	74	Down
3	TARID	68	Down
4	SRP9	66	Down
5	AF165147	66	Down
6	AL024497	64	Down
7	AC103702	62	Down
8	TMTC1	60	Down
9	LINC01535	58	Down
10	AC073115	52	Down

### 3.5 Target gene prediction of lncRNA

Furthermore, cis-regulation predictive analysis on the differentially expressed lncRNAs and mRNAs was performed (Figure 7B). At the same time, according to the correlation coefficient, the ranking was carried out (Table 3). Comparing the results of prediction with the above 10 differentially expressed lncRNAs, it was found that FGF7 and HOXB4/5/6 might play an important role in the occurrence and development of CPT as targets of FAM227B and AC103702. These interactions between lncRNAs and mRNAs, as the possible mechanism of CPT, might become potential molecular targets for CPT therapy.

**TABLE 3:** Cis-regulation predictive analysis on the differentially expressed lncRNAs and mRNAs was performed according to the correlation coefficient.

LncRNA	mRNA	Pearson
AC103702	HOXB4	0.85
	HOXB5	0.98
	HOXB6	0.71
FAM227B	FGF7	−0.96
CTD-2201I18	THBS4	0.94
AC004080	HOXA13	0.79
HOXA11-AS		0.54
AL391056	NTMT1	0.69
PVT1	MYC	0.68
EMX2OS	EMX2	0.66
MIR22HG	WDR81	−0.46

### 3.6 qRT-PCR

**FIGURE 8:**
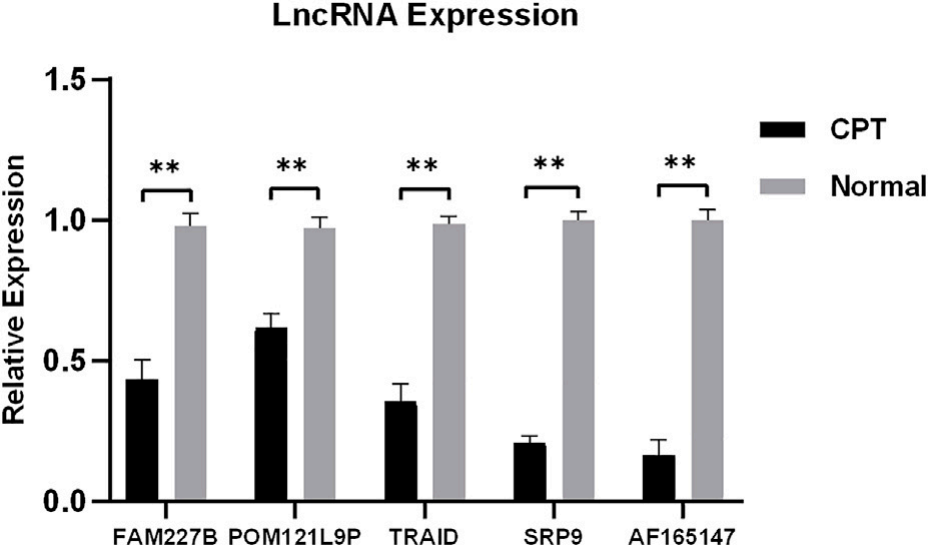
Five differentially expressed lncRNAs were selected for qRT-PCR. qRT-PCR, quantitative reverse transcription-polymerase chain reaction.

To verify the reliability of microarray, five differentially expressed lncRNAs were selected for qRT-PCR. The results showed that the expression of FAM227B, POM121L9P, TARID, SRP9, and AF165147 were downregulated (*p* < 0.05), which were consistent with microarray (Figure 8).

## 4 Discussion

In this study, high-throughput microarray sequencing technology was used to identify the genome of periosteal stem cells from normal and CPT patients, detect the differentially expressed lncRNAs and mRNAs, and statistically analyze their biological functions.

Since CPT has been reported, its pathogenesis remains to be unclear (BOYDHarold, 1982; Pannier, 2011). Through previous epidemiological and pathological studies, some scholars have found that 40%–80% of CPT patients are NF-1 gene mutation carriers, and there are neurofibroma-like tissues growing and invading in the diseased periosteum (Carlier et al., 2016; Van Royen et al., 2016). Histological studies have also found that the periosteum of the lesion site of CPT has abnormal thickening (Hermanns-Sachweh et al., 2005; Pannier, 2011), accompanied by fibrous tissue hamartoma-like hyperplasia, which was similar to the pathology of NF-1 patients (Van Royen et al., 2016). We also found in the previous study that the exosomes of CPT patients with NF-1 can significantly inhibit bone formation and stimulate bone absorption, thereby reducing the bone mass of trabecular and cortical bone in patients (Yang et al., 2021). However, some scholars suggested that whether CPT is combined with NF-1 has no significant difference in clinical characteristics, histopathology and postoperative tibial healing (Guanghui et al., 2019). At the same time, some scholars believed that the local bone vasculature disorder caused by the abnormal periosteal blood vessels in CPT is also one of the important factors for the formation of pseudarthrosis. The critical role of the vasculature in bone health has been repeatedly demonstrated in previous studies (Chen et al., 2021; Charlotte and Anjali, 2022). Benita et al. found that the periosteal arteries of the lesion were occluded, resulting in decreased osteogenic capacity in CPT patients (Hermanns-Sachweh et al., 2005). Tommi et al. confirmed that the vessel wall at the pseudarthrosis was thickened, the lumen was narrowed, and the local blood supply was blocked (Tommi et al., 2006).

With the continuous progress of molecular biology technology in recent years, our understanding of the pathogenesis of CPT is also deepening. Lei et al, (2001) found that there is excessive proliferation of fibroblasts and abnormal expression of related cytokines in periosteum of CPT lesions, which can result in abnormal thickening of periosteum, suggesting that CPT may be a disease originated from periosteum. Periosteum is composed of three layers: fibrous layer, undifferentiated layer and germinal layer. The fibrous layer is mainly composed of collagen fibers. The germinal layer is rich in PSCs, osteoblasts, mononuclear macrophages and other cells, with good osteogenic ability (Lin et al., 2014). The balance of bone metabolism is maintained by osteoblasts and osteoclasts, which are differentiated from PSCs under specific conditions. Studies have found that compared with normal children, diseased PSCs from CPT patients show lower osteogenic differentiation ability and faster aging speed (Cho et al., 2008; Bajada et al., 2009; Granchi et al., 2012). Some scholars have used stem cells in the treatment of local CPT and achieved good curative effect (Tikkanen et al., 2010). These results suggest that the periosteal lesions of CPT are caused by abnormal PSCs to some extent.

In recent years, high-throughput transcriptome sequencing technology has been increasingly recognized by scholars due to its fast speed, high throughput and good accuracy. By comparing and analyzing non-coding RNA such as lncRNA and miRNA between diseased and normal cells, key genes and regulatory pathways related to diseases can be screened out, which is one of the mainstream methods to explore the pathogenesis of diseases at present. Many studies have shown that non-coding RNA plays an important role in the differentiation and regulation of stem cells such as osteogenesis and adipogenesis. Zhuang et al. (Zhuang et al., 2015) sequenced the stem cells from ankylosing spondylitis and normal patients and found that Lnc ZNF354A-1, Lnc LIN54-1, Lnc FRG2C-3 were involved in abnormal osteogenic differentiation of MSCs in ankylosing spondylitis patients. Song et al. (Song et al., 2015) induced the osteogenic differentiation of BMSCs and found that there was a complex interaction between 217 lncRNAs and miRNAs, which affected the osteogenic differentiation of BMSC by regulating ECM receptor, adhesion spot and other pathways. Huang et al. (Huang et al., 2022) found that differentially expressed mRNA was mainly downregulated in CPT MSCs, which was related to the decline of osteogenic differentiation ability of MSCs. However, there are few studies on lncRNA in the pathogenesis of CPT. Therefore, by exploring the regulatory mechanism of lncRNA-mRNA in the occurrence and development of CPT, it may provide potential targets for the prevention and treatment of CPT. In this study, lncRNA-mRNA were identified in five pairs of normal and CPT PSCs by high-throughput microarray sequencing technology, and differentially expressed lncRNAs and mRNAs were screened out. The results showed that there were 194 differentially expressed lncRNAs (73 upregulated genes and 121 downregulated genes), and 822 differentially expressed mRNAs (311 upregulated genes and 511 downregulated genes). Both lncRNAs and mRNAs were mainly downregulated in CPT PSCs, which may be closely related to the decline of osteogenic differentiation ability and abnormal bone metabolism.

The enrichment analysis of differentially expressed mRNAs showed that the biological functions with the most significant differences were mainly concentrated in skeletal system development, tissue morphogenesis and NABA core matrisome. Signaling pathway analysis showed that the pathways with the most significant differences were mainly concentrated in pathways in cancer, PI3K-Akt signaling pathway and cytokine-cytokine receptor interaction, which was similar to the previous literature (Song et al., 2015). Moreover, through PPI enrichment analysis, we found that the function of differentially expressed mRNA regulatory proteins was related to skeletal system development, tissue morphogenesis, MAPK signaling pathway regulation, etc. The results further showed that the development and construction of skeletal system plays an important role in the pathogenesis of CPT.

LncRNA-mRNA co-expression network can directly show the interaction of the whole transcription level. According to the position of genes in the co-expression network, the key genes can be efficiently inferred and screened out, which may play an important role in the pathogenesis. In this study, a series of key genes, such as FAM227B, POM121L9P, AF165147, and AC103702, were listed by analyzing the differences of co-expression network and the changes of connectivity degree abundance. These 10 differentially expressed lncRNAs had the highest connectivity degree and were located in the center of the co-expression network, which means some of them might be closely related to the pathogenesis of CPT. Interestingly, these key lncRNAs were all downregulated in CPT PSCs. This was also consistent with the results of enrichment analysis mentioned above. At the same time, we predicted the targets of differentially expressed lncRNAs and ranked them according to the correlation coefficient. The results showed that FAM227B-FGF7 and AC103702-HOXB4/5/6 were potential target pathways with clinical significance. Homologous (HOX) gene is a relatively evolutionarily conserved transcription factor, which plays an important regulatory role in embryonic development (Deschamps and and van Nes, 2005). Studies have shown that HOX is a potential mechanism to maintain the tripotency of PSCs, and inhibition of HOX expression will lead to the loss of tripotency in stem cells (Jacopo et al., 2012). Bradaschia et al. found that HOX-positive periosteum contains more PSCs and shows better differentiation ability (Bradaschia-Correa et al., 2019). Liu et al. found that HOXB7, as the downstream target of miR-24, can block the inhibition of miR-24 on osteogenic differentiation of MSCs (Huang et al., 2022). FGF7 is a regulator of interstitial cell secretion, and its combination with FGFR plays an important role in regulating cell growth, migration and differentiation (Pastar et al., 2014; Liu et al., 2020). Previous studies have found that FGF7 can improve bone formation and promote osteogenic differentiation of stem cells (Poudel et al., 2017; Liu et al., 2018). Jeon et al. (2013) found that FGF7 can induce osteogenic differentiation of embryonic stem cells through ERK/Runx2 signaling pathway. Qiu et al. (2022) reported that over-expression of miR-381-3p inhibits osteogenic differentiation of BMSCs by targeting negative regulation of FGF7. All the above studies indicated that HOX and FGF7 genes play a positive role in the process of cell osteogenesis. In this study, it was found that both FAM227B and AC103702 showed a downregulation trend in CPT PSCs, while their downstream targets FGF7 and HOXB4/5/6 were also at low expression levels. It is reasonable to speculate that these two signaling pathways are closely related to the decline of osteogenic differentiation ability of CPT PSCs. Of course, the function of lncRNA/mRNA in CPT PSCs still remains largely unknown and needs further study.

There were still some limitations in this study. First of all, the sample size included in this study was small, so there might be some certain sample heterogeneity. The results could not fully represent the characteristics of all patients and need to be verified by larger samples in the future. Secondly, the basic data of patients could not be homogenized, so patients of different ages and types might have different gene expressions. Finally, on the basis of bio-informatics analysis, we need to further verify the function in animal experiments in the future, and further study the role of lncRNA-mRNA in the occurrence and development of CPT, so as to provide new ideas and scientific basis for the prevention and treatment of CPT.

In this study, the differential expression of lncRNAs and mRNAs in normal and CPT PSCs was detected by high-throughput sequencing analysis. The biological processes and signaling pathways of some differentially expressed lncRNAs and mRNAs were related to skeletal development and construction. In addition, by exploring lncRNA-mRNA co-expression and lncRNA target prediction, two significantly correlated lncRNA-mRNA signaling pathways were screened out. These results were helpful to deepen our understanding of the pathogenesis of CPT and provide potential therapeutic targets.

## Data Availability

The original contributions presented in the study are publicly available. This data can be found here: https://www.ncbi.nlm.nih.gov/geo/query/acc.cgi?acc=GSE200069

## Abbreviations

PSCs, periosteal stem cells; CPT, congenital pseudarthrosis of the tibia; lncRNA, long-chain non-coding RNA; mRNA, messenger RNA; GO, Gene ontology; KEGG, Kyoto encyclopedia of genes and genomes; PPI, Protein -protein interaction; MCODE, Molecular Complex Detection; HOX, Homologous; NF-1, Type I neurofibromatosis.
