# Supplementary material for: Differential expression and effect analysis of lncRNA-mRNA in congenital pseudarthrosis of the tibia
Source: Front Genet. 2023 Feb 6;14:1094298. doi: 10.3389/fgene.2023.1094298 (PMC9939773; doi:10.3389/fgene.2023.1094298)
Supplement: Supplementary file 1 [file Table1.DOCX]

| **Network** | **Annotation** |
| --- | --- |
| MCODE-ALL | GO:0001501\|skeletal system development\|-12.9; GO:0007389\|pattern specification process\|-10.5; M5884\|NABA CORE MATRISOME\|-10.2 |
| MCODE_1 | GO:0048704\|embryonic skeletal system morphogenesis\|-10.1; GO:0048706\|embryonic skeletal system development\|-9.5; GO:0009952\|anterior/posterior pattern specification\|-8.6 |
| MCODE_2 | R-HSA-3656225\|Defective CHST6 causes MCDC1\|-14.7; R-HSA-3656243\|Defective ST3GAL3 causes MCT12 and EIEE15\|-14.7; R-HSA-3656244\|Defective B4GALT1 causes B4GALT1-CDG (CDG-2d) \|-14.7 |
| MCODE_3 | R-HSA-416476\|G alpha (q) signalling events\|-8.6; R-HSA-373076\|Class A/1 (Rhodopsin-like receptors) \|-7.8; hsa04080\|Neuroactive ligand-receptor interaction\|-7.7 |
| MCODE_4 | WP5087\|Malignant pleural mesothelioma\|-7.4; GO:0043410\|positive regulation of MAPK cascade\|-7.1; GO:0043408\|regulation of MAPK cascade\|-6.6 |
| MCODE_5 | GO:0060485\|mesenchyme development\|-6.3; GO:0048729\|tissue morphogenesis\|-5.2 |
